# Editorial: Challenges in eating behavior

**DOI:** 10.3389/fpsyg.2025.1563234

**Published:** 2025-02-11

**Authors:** Nicholas T. Bello, C. Alix Timko, Tuyen Van Duong, Edward A. Selby

**Affiliations:** ^1^Department of Animal Sciences, School of Environmental and Biological Sciences, Rutgers, The State University of New Jersey, New Brunswick, NJ, United States; ^2^New Jersey Institute for Food, Nutrition and Health, Rutgers, The State University of New Jersey, New Brunswick, NJ, United States; ^3^Department of Psychiatry, Perelman School of Medicine, University of Pennsylvania, Philadelphia, PA, United States; ^4^Department of Child and Adolescent Psychiatry and Behavioral Sciences, Children's Hospital of Philadelphia, Philadelphia, PA, United States; ^5^International Master/Ph.D. Program in Medicine, College of Medicine, Taipei Medical University, Taipei, Taiwan; ^6^School of Nutrition and Health Sciences, Taipei Medical University, Taipei, Taiwan; ^7^Department of Psychology, School of Arts and Sciences, Rutgers, The State University of New Jersey, Piscataway, NJ, United States

**Keywords:** binge eating disorder, meat, eating rate, social media and marketing strategy, bite

Eating is a complex human behavior and a core component of lifestyle medicine. Given the ever-changing influences of an interconnected society, new challenges further complicate our perceptions, food choices, and motivations to eat. The goal of this Research Topic was to examine the challenges in eating behavior research and practice. The five articles in this Research Topic represent a wide range of challenges including characterizing the unhealthy food behaviors in Indian adolescents, socioeconomic meat consumption patterns, body recognition challenges in people with binge eating disorder (BED), standardization of automatically assessing bite counts in meal events, and determining the role of humor on “healthy” food posts on social media.

Adolescence is a critical developmental stage for establishing adult eating behaviors (Daly et al., 2022). Jena et al. examined the regional differences of unhealthy food behavior in India, which has one of the world's largest populations of adolescents. Using a scoping review framework of the available literature, the authors reviewed the data from 33 articles to discuss the differences in knowledge and perceptions of unhealthy foods, unhealthy food practices, and factors influencing unhealthy food choices. Most of the studies (23 out 33) provided sex-stratified results based on unhealthy food choices with a predominance of the studies representing the Southern zone of India (13 out of 33). The authors discuss the significant challenges of unhealthy food choices among Indian adolescents and the recommendations for addressing these challenges.

Animal protein or meat is a readily available food source of essential amino acids and micronutrients in the human diet (Massey, 2003). Societal meat consumption patterns are dependent on interrelated economic, environmental, and cultural factors (Petersen and Hirsch, 2023). Therefore, establishing sustainable practices to address the consumer demand for meat is often a challenge for the industry, policy makers, and regulatory agencies. One strongly influencing consideration is related to whether a country has a *developed* or *emerging* economy. Delley et al. surveyed random populations of people in a developed country, Switzerland (n = 643 participants), and emerging country, Vietnam (n = 616 participants), to profile and segment meat consumption patterns. Cluster analysis of the sampled population indicated five segments with three segments common to both Switzerland and Vietnam. These included meat lovers (20% in Switzerland vs. 19% in Vietnam), proactive (22% in Switzerland vs. 14% in Vietnam), and suggestible (19% in Switzerland vs. 25% in Vietnam). Not common segments were traditional (19%) and basic (20%) in the Swiss population, whereas confident (16%) and anxious (26%) were indicated in the Vietnamese population. The results were reported by cluster and country for diet type (Omnivores vs. Flexitarians), average weekly meat consumption, food choice importance, and socio-demographic factors. Practical implications for implement meat reduction strategies per segment and countries are discussed, as well as possible educational and regulatory measures.

Using reflexive thematic analysis, Olsen et al. examined the relationship of body experiences and BED. On the last day of inpatient treatment for BED, six women (25–50 years old) consented to participate in a semi-structured in-depth interview (45–90 min) regarding body, food, social relations and treatment experiences. Interviews were conducted within 3 months after discharge. The collected interviews were subjected to a six phase reflexive data analysis by the authors and resulted in three meta-themes. These themes were: *Relational challenges and feeling could not be talked about at home, Contempt for body image disturbs the experience of self and others*, and *Their body has not been a theme in previous treatment*. Olsen et al. discuss these meta-themes and related themes in BED psychopathology. Additionally, there is an emphasis on the challenge of recognition of body experience in therapy and “living with BED in a big body”.

Individual meal events, such as eating activity or chewing bouts, are informative metrics for assessing eating behaviors. Several challenges are that current laboratory-based methods for assessing meal-related eating behavior relies on investigator-based visual assessments, detailed manual annotations of meals, and ensuring consistency between individual annotators (Tufano et al., 2022). These methods are time consuming and prone to subjectivity. To address these issues, Tufano et al. developed an automated processing of the threshold for facial key points and rule-based criteria to count bites. The objective of the study was to assess the performance of the automated processing with manual annotations of meal video recordings from 15 participants. The automated system varied from 36.6% to 94.6% accuracy compared with manual annotation and it performed consistently across food textures. The authors discuss the potential opportunities for future research applications.

Social media influences food choices (Mc Carthy et al., 2022). In a brief research report Reijnen et al. explored the effect of humor on the intention of purchasing of healthy foods on a social media platform. The study design used four embedded jokes posts (2 of the 4, explicitly included the word “fat”) and six non-joke filler posts, each post contained a repost and emoji-like slider option in an artificial Instagram feed. The influence of the joke post on the willingness to buy the subsequent healthy food post was tested in the study participants (*n* = 411; mean age 24.19 ± 4.24 yrs old, 70.6% female). Results indicated a relationship between joke type and intention to buy recognized healthy food options. Purchase intent was decreased when the word “fat” was used in the joke post. The authors further discuss the influence of humorous or non-humorous on food choices.

Overall, the mix of original research articles, reviews, and brief reports in this Research Topic represent the varied challenges in the field of eating behavior. Eating behavior will continue to be a dynamic and intriguing area, even as we continue to make progress in understanding the biological, psychological, and social underpinnings of eating. Identifying and addressing challenges, such as those represented here, will be crucial for the ongoing tasks of destigmatizing disordered eating, promoting healthy lifestyles, and developing effective therapeutic options.
